# Supplementary material for: Stacking Model‐Based Classifiers for Dealing With Multiple Sets of Noisy Labels
Source: Biom J. 2025 Mar 12;67(2):e70042. doi: 10.1002/bimj.70042 (PMC11898607; doi:10.1002/bimj.70042)
Supplement: Supplementary file 1 — Supporting Information [file BIMJ-67-e70042-s001.zip › density-based-ensemble-model-main/competitors_real_data/netlab-master/netlab-master/help/dem2ddat.htm]

Netlab Reference Manual dem2ddat


# dem2ddat

## Purpose

Generates two dimensional data for demos.

## Synopsis

```
data = dem2ddat(ndata)
```

```
[data, c] = dem2ddat(ndata)
```

## Description

The data is
drawn from three spherical Gaussian distributions with priors 0.3,
0.5 and 0.2; centres (2, 3.5), (0, 0) and (0,2); and standard deviations
0.2, 0.5 and 1.0. `data = dem2ddat(ndata)` generates `ndata`
points.

`[data, c] = dem2ddat(ndata)` also returns a matrix containing the
centres of the Gaussian distributions.

## See Also

`demgmm1`, `demkmean`, `demknn1`

---

**Pages:**
Index


---

Copyright (c) Ian T Nabney (1996-9)
